# Aubergine stem restores reproductive damages following diabetes mellitus induction in male mice

**DOI:** 10.1002/fsn3.3767

**Published:** 2023-10-21

**Authors:** Parsa Rostamzadeh, Vahid Shokri‐Asl, Fatemeh Mansouri Torghabeh, Samira Davoudi, Ahmad Haghzadeh, Shima Moradi

**Affiliations:** ^1^ Department of Anatomical Sciences, Medical School Kurdistan University of Medical Sciences Sanandaj Iran; ^2^ Department of Reproductive Biology, Faculty of Advanced Medical Sciences Tabriz University of Medical Sciences Tabriz Iran; ^3^ Department of Physiology Sciences, Medical School Mashhad University of Medical Sciences Mashhad Iran; ^4^ Student Research Committee, Faculty of Dentistry, Tabriz Branch Islamic Azad University Tabriz Iran; ^5^ Department of Anatomical Sciences, Medical School Kermanshah University of Medical Sciences Kermanshah Iran

**Keywords:** apoptosis, Aubergine, diabetes mellitus, male reproduction, testis

## Abstract

Diabetes mellitus unbalances cellular antioxidant levels. This phenomenon can potentially lead to cellular damage and apoptosis in the male reproductive system. Besides, herbal‐based antioxidants can prevent these detrimental changes. Thus, we assessed the probable role of Aubergine stems with antioxidant and anti‐hyperlipidemic characteristics on reproductive damage following diabetes mellitus induction. Forty male NMRI mice were categorized into groups of control and treatments. Diabetes was induced by a single dose of streptozotocin (60 mg/kg), and the extract was administered at various doses (100, 300, and 500 mg/kg) daily for 4 weeks. Antioxidative features of the extract were approved by phytochemical assays and ferric‐reducing ability of plasma. Side‐effects of diabetes were also assessed by the malondialdehyde (MDA) and Griess techniques. Sperm parameters, LH, FSH, and testosterone levels, the TUNEL assay, histopathologic alteration, and apoptotic genes (p53, caspase‐3, Bcl‐2) were evaluated. Results showed that diabetes increased oxidation levels and the extract accelerated total antioxidant capacity status. Sperm parameters and hormone levels were restored following extract administration in diabetic animals. Also, the apoptosis rate decreased following extract administration in diabetic animals. We concluded that diabetes can elevate the levels of oxidation and suppress the antioxidant power. These pathologic changes were restored by Aubergine stem, leading to decreased levels of apoptosis and normal serum levels of testosterone, LH, and FSH.

## Graphical abstract

Diabetes mellitus represents adverse effects on the antioxidant homeostasis of spermatogenic cell lines. The root of the Aubergine stem is rich in natural antioxidant substances. The root of the Aubergine stem aids in the restoration of Leydig cells and physiological levels of testosterone and inhibits the apoptosis of the spermatogenic lineage.

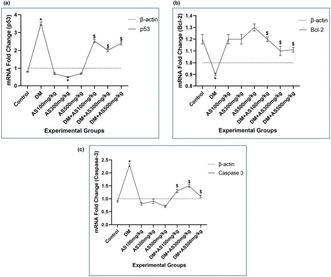

## 1 INTRODUCTION

Reproduction is a biological process in which a single‐cell organism is generated through fertilization among male and female gametes. Unhealthy gametes cannot lead to successful fertilization. The health status of gametes is affected by some pathological variations, like diabetes mellitus (DM) (Ritchie & Ko, 2021). The testis is the male reproductive organ that is involved in spermatogenesis through spermatogenetic cell lines and the production of testosterone (Tes) by healthy Leydig cells. The physiological function of spermatogenic lineage can lead to healthy sperm. This process completely depends on the normal serum levels of Tes (Gunes & Esteves, 2021). According to various structural features of sperms, such as a decreased amount of sperm cytoplasm and the presence of a small amount of intracellular antioxidant components, these cells are seriously vulnerable to reactive oxygen specious (ROS) attack, lipid peroxidation, and cellular oxidative stress. In these situations, the DNA damage to sperm can lead to cell apoptosis (Agarwal et al., 2018).

Pathophysiologically, DM is considered a metabolic disorder caused by high levels of blood sugar (BS). This pathologic condition can induce various lethal cellular fluctuations, such as cell destruction and imperfect cell function, especially in the reproductive system. Numerous articles have reported an increase in oxidative stress levels following the onset of DM. Components of the reproductive system are affected and damaged by DM, which can eventually lead to male infertility (Maresch et al., 2018).

Herbalism is the study of the pharmacognosy and application of medicinal plants for human diseases. Today, medicinal plants have received a lot of attention due to their natural therapeutic features with less side effects. Aubergine, or egg‐plant, with the scientific name *Solanum melongena* is a tropical plant with therapeutic features. According to the published studies, the stem of this plant, located in the upper part, represents anti‐lipidemic and antioxidant characteristics. This part of the plant is also used to relieve rheumatic pain, hypercholesterolemia, and skin pathologic conditions (Yarmohammadi et al., 2021). The therapeutic features of *Solanum melongena* are totally associated with the presence of various major compounds, including chlorogenic acid, nasunin (a unique anthocyanin with potent antioxidant activity), delphinidin (a type of anthocyanin with potential health benefits), solamargine and solasonine (steroidal alkaloids with cytotoxic effects on cancer cells and anticancer properties), kaempferol (a flavonoid with antioxidant and anti‐inflammatory properties), quercetin (a flavonoid with antioxidant and anti‐inflammatory effects), rutin (a flavonoid with neuroprotective effects), and beta‐carotene (precursor of vitamin A with antioxidant properties) (Laulloo et al., 2021).

Since DM reduces male fertility levels by inducing cellular oxidative stress in testicular tissue, the application of plant‐based antioxidants can probably increase the oxidative power of cells against the detrimental effects of DM. Thus, in the present study, we aimed to evaluate the probable anti‐oxidative and anti‐lipidemic effects of Aubergine stem (AS) on male reproductive parameters of diabetes‐induced male mice.

## 2 MATERIALS AND METHODS

### 2.1 Experimental animals

NMRI male mice (22–28 g, 8–12 weeks) were kept in an animal house for 2 weeks for adaptation to the new environment. All standard settings including 12 h light/12 h dark, 22 ± 2°C, 50%–60% humidity, and free access to food/water were totally provided. The experimental protocols were conducted in accordance with ethical principles and under the supervision of the University's Ethics Committee.

### 2.2 Study groups and animal/organ weighing

Forty animals were divided into 8 groups (*n* = 5): 1st, control; 2nd, DM; 3rd–5th, three doses of AS (100, 300, and 500 mg/kg); and 6th–8th, DM + AS 100, 300, and 500 mg/kg. Animals in the control group received 0.2 mL of distilled water, and DM was induced by a single intraperitoneal (IP) injection of streptozotocin (STZ, 60 mg/kg). Also, the body weight and the testis weight were recorded in two stages: prior to the experiment and prior to the tissue sampling.

### 2.3 Chemicals, drugs, and laboratory kits

All chemicals were purchased from Sigma Aldrich Co., including STZ (CAS NO. 18883‐66‐4), ethanol (CAS NO. 64‐17‐5), Whatman filter paper (Whatman® qualitative filter paper, Grade 1), vacuum pump (Chemat vacuum pumps, KW‐4AVP‐115 V), formaldehyde (CAS NO. 50‐00‐0), DMEM/Nutrient Mixture F‐12 Ham (MDL NO. MFCD00217417), eosin staining (CAS NO. 15086‐94‐9), nigrosine staining (CAS NO. 8005‐03‐6), FRAP assay kit (MAK369), ELISA kit for Tes measurement (CAS NO. T1500), ELISA kit for NO measurement (CAS NO. 23479), malondialdehyde (MDA) kit (CAS NO. MAK085), Apo‐Direct TUNEL Assay Kit (CAS NO. APT110). In this study, a light microscope was used (Leica RM 2125; Leica Microsystems Nussloch GmbH). RNA was extracted by the QIAGEN RNA purification mini kit. Spectrophotometer machine (UV1240; Shimadzu) was used for assessment of RNA quality. DNA was synthetized using BioFact RT Series, Korea, and gene expression was assessed using High ROX BioFact™ 2X Real‐Time PCR Smart mix SYBR Green PCR master mix.

### 2.4 Herbal extract preparation

According to Choi's protocol, following the preparation of the eggplant from the phytosanitary center, the AS was cut and dried in the shade for 1 week. AS powder was prepared and dissolved in boiling water (2 L) for 2 h. Then, the resulting solution was dissolved in 96% alcohol (with an extract/alcohol ratio of 1:5). The solution was incubated in 4°C refrigerator for 3 days. Then, the precipitation was discarded, and the solution was centrifuged (twice, 4000 r.p.m., 15 min). Finally, the remaining solution containing the herbal extract was placed in an incubator (37°C) for a week. After the evaporation of the hydro‐alcoholic solution, the remaining precipitation was separated, weighed as a plant extract, and stored for future therapeutic purposes (Choi et al., 2016).

### 2.5 Dose calculation

The OECD guideline was hired for the analysis of lethal doses of chemicals. Four main doses of AS were determined, including 100, 300, 500, and 1000 mg/kg. This extract was administered orally for 4 weeks to four groups of animals (*n* = 3 in each testing group). The deaths of animals in each group were recorded. According to the protocol, the death >1 animal in each group was reported as a toxic dose. Finally, non‐lethal doses were defined and used in the experiment (Saha et al., 2017).

### 2.6 Semi‐quantitative phytochemical screening

Five gram of AS powder was dissolved in 96% ethanol (1 L). Then, for thin‐layer chromatography (TLC) identification, silica gel with 0.2 mm thickness was hired. The ambulatory phase for terpenes and sterols was also prepared with the use of Liebermann–Burchard reagent and hexane/ethyl acetate. After the classical procedure of acid/base extraction, the TLC analysis of alkaloids was done. TLC was developed in n‐butanol/acetic‐acid/water for flavonoids detection, and an aluminum chloride solution (1%) dissolved in methanol (366 nm of UV) was provided for spot visualization. Gelatin solution (1%) and froth tests were also used for tannin and saponin detection, respectively. Potassium hydroxide (10%, dissolved in methanol) was used for the identification of anthraquinones and phlobatannins (Roshankhah et al., 2020).

### 2.7 Induction of DM

Citrate acid (1.05 g) was combined with sodium citrate (1.48 g) in dH_2_O (100 mL) to form citrate buffer (0.1 M) at pH 4.5. Weighted STZ was dissolved in cold citrate buffer and followed by sterilization (using a 22 μm pore filter). Prior to DM induction, the BS was assessed, and only the animals with BS levels ranging between 80 and 100 mg/dL were considered as healthy animals. DM was induced by the IP injection of STZ at a dose of 60 mg/kg. Then, for confirmation of successful DM induction, the BS factor was also measured, and the animals with BS > 250 mg/dL were considered diabetic animals and entered the study (Akbarzadeh et al., 2007).

### 2.8 Route of administration of Aubergine stem

The extract treatment was applied daily at 10 a.m. for 4 weeks. In this procedure, the extracts (with various doses of 100, 300, and 500 mg/kg) were dissolved in 1 mL of distilled water and prescribed orally.

### 2.9 Animal dissection and sampling

A day after the last treatment, the animals were given a light sedation (though 50IU of ketamin10IU/xylazin90IU, IP injection) and then sacrificed through cervical dislocation. A laparotomy was used to access the spermatic cord. Testes were found and dissected. The right testis was fixed in formaldehyde at 10% and the left one was loaded in liquid nitrogen for gene expression assessments. The epidydimis was also dissected in DMEM/F10 condition media as a sperm‐releasing solution. Finally, the thoracotomy procedure was applied, and the blood was aspirated from the right ventricle for biochemical analysis (Roshankhah et al., 2020).

### 2.10 Sperm parameter assessments

For this purpose, sperm samples were collected, and viability, motility, count, and normal morphology were assessed as sperm parameters. The eosin stain was used for viability assessment. Through the penetration of eosin into the intracytoplasmic matrix of dead cells, the distinction between living and dead cells was determined. 20 μL of semen fluid was mixed with an equal volume of eosin stain solution. 2–5 min later, the solution was poured onto a neobar culture slide, and at least 100 sperm cells were counted from each random sample according to the 10 view fields of imagining, and the percentage of living sperm cell was documented. Only progressive motility was considered as normal sperm movement. The movement of sperm in a straight line or large circles was defined as progressive sperm motility. To analyze the number of sperm cells, 400 μL of the sperm suspension was diluted with formaldehyde fixative. Approximately 15 μL was transferred from the diluted solution into a hemocytometer by a Pasteur pipette. The hemocytometer was located into a Petri dish with dampened filter paper and allowed to stand for 10 min. The stable sperm were counted and assessed per 250 small squares of the hemocytometer using a ×40 objective. The number of sperm per mm^3^ was calculated by the formula “number of counted sperm × dilution/number counted (in mm^2^) × depth of the chamber”. Normal sperm morphology was assessed through the examination of sperm smears. Eosin/nigrosin staining was used to estimate the morphology of normal spermatozoa (400×) in the field of head and tail irregularities (Cooper et al., 2010).

### 2.11 Ferric‐reducing ability of plasma (FRAP) assay

Through this assay, the concentration of cellular antioxidants can be determined. In this reaction, Cu^2+^ is converted to Cu^+^ by small molecules and proteins. The reduced Cu^+^ chelates with a colorimetric probe, giving a broad absorbance peak at 570 nm, which is proportional to the total antioxidant capacity. Antioxidant capacity was donated as Trolox equivalent as a vitamin E analog. Trolox standards were provided, and the samples (100 μL) were also obtained and added to the wells. 100 μL of Cu^2+^ working solution were added to all standards and sample wells. They were incubated for 90 min, and absorbance was measured at 570 nm (Henríquez et al., 2008).

### 2.12 Serum levels of Tes, LH, and FSH

Hormone levels (Tes, LH, and FSH) as an important index representing the levels of Leydig cells’ activity and hypothalamic–pituitary–gonadal axis were measured from blood serum and examined through the ELISA kit. 25 μL of each standard, control, and sample were dispensed into the appropriate wells, and 200 μL of enzyme conjugate was poured into each well. The solution was incubated at room temperature for 60 min. Then, the wells were rinsed with washing buffer (400 μL) for three times. Substrate solution (200 μL) was added to the wells and incubated for 15 min at room temperature. Then, the stop solution (100 μL) was added, and the optical density (OD) of each solution (LH, FSH, and Tes) was calculated at 450 nm (Li et al., 2020).

### 2.13 Griess technique

Nitrite oxide (NO) was measured by Griess assay according to the routine protocol. In this procedure, equal volumes of 1× Griess reagent and the samples were mixed. 10 min later, the absorbance was read at 450 nm (Möller et al., 2019).

### 2.14 Malondialdehyde (MDA) measurement

Thiobarbituric acid reactive substances (TBARS) were formed as a byproduct of lipid peroxidation following the degradation of lipids in the cell membrane. TBARS can be detected by an MDA assay based on the colorimetric analysis. This index represents the levels of oxidative stress that damage cell structures. In this procedure, 600 μL of TBA reagent was added into each well containing 200 μL of standard and 200 μL of sample. The wells were incubated for 60 min at 95°C. 200 μL of the reaction mix was added to each well, and following 15 min, the optical density was assessed at 450 nm (Jentzsch et al., 1996).

### 2.15 Histopathological alterations

For assessment of histopathological changes, routine histological protocols were followed and the slides were assessed under a routine microscope (400×). In this procedure, the fixed tissue samples were treated using a tissue processor machine. Then, the paraffin‐embedded tissue was cut (5 μm) and stained using the H&E technique. Many histopathological changes were assessed, including the diameter of seminiferous tubules, the thickness of the germinal epithelium, the quantification of seminiferous tubules, and Leydig cell counting. For all assessments, 100 sections of each treatment group were analyzed and the mean number was reported. Images were analyzed using a computerized image processing and analysis program (Bab Bs200Pro) (Kazemi et al., 2016).

### 2.16 In situ direct DNA fragmentation (TUNEL) assay for apoptosis detection

According to the protocol of Fazlioglu, the TUNEL assay was hired for the detection of DNA fragments. Apoptotic index (AI) was defined as the total apoptotic cells count per seminiferous tubule to assess the rate of apoptotic cells. Briefly, in this assay, the samples were fixed using paraformaldehyde at 1%. After tissue section preparation, they were treated with a staining solution, and finally, the slides were assessed microscopically (Fazlioglu et al., 2008).

### 2.17 RNA extraction and Real‐Time quantitative PCR

All laboratory gene expression assessments were conducted based on the protocol and manufacturer's instructions, and practical manipulation was applied according to Salahshoor's protocol. The expression of caspase‐3 (F: CTGGACTGTGGCATTGAGAC, R: CAAAGGGACTGGATGAACC), Bcl‐2 (F: GATGCCTTTGTGGAACT, R: GAGACAGCCAGGAGAAATCA), and p53 (F: AGAGACCGCCGTACAGAAGA, R: GCATGGGCATCCTTTAACTC) genes was evaluated. Primers were designed (Oligo software), and the sequences were blasted into the NCBI database. β‐actin (F: GGCACCACACCTTCTACAATG, R: GGGGTGTTGAAGGTCTCAAAC) was used as a housekeeping gene. Finally, gene expression levels were measured using the Ct (2^−ΔΔt^) method (fold changes) (Salahshoor et al., 2018).

### 2.18 Statistical analysis

In order to assess data normality, the Kolmogorov–Smirnov test was applied. A one‐way analysis of variance (one‐way ANOVA) was used for statistical analysis, and the Tukey's post hoc test was used to determine the difference between the groups. SPSS (IBM® SPSS® software, version 16) was used for data analysis, and the results were expressed as mean ± SD. *p <* .05 was also considered a significant level.

## 3 RESULTS

### 3.1 Phytochemical analysis of AS

The AS extract was completely rich in saponins, flavonoids, and tannins. Also, phytochemicals, including alkaloids, phlobatannins and anthraquinones, were present with a lower percentage (Table 1).

**TABLE 1:** Phytochemical screening of hydroalcoholic extracts of Aubergine stem.

Phytochemical assay	Extract
Saponins	+++
Flavonoids	+++
Alkaloids	+
Tannins	+++
Anthraquinones	+
Phlobatannins	+

*Note*: +++ and + indicate strong and weak intensity reactions, respectively. Data were analyzed using semi‐quantitative phytochemical screening and are presented as negative or positive markers.

### 3.2 Dose calculation

Evaluations based on the OECD protocol showed that no animal deaths were reported in groups treated with various doses of 100, 300, and 500 mg/kg of AS. Only the dose of 1000 mg/kg of AS represented animal death (2 out of 3 animals). Thus, a dose of 1000 mg/kg of extract was considered a toxic dose; besides, triple doses of 100, 300, and 500 mg/kg were used to evaluate the probable therapeutic effects.

### 3.3 Sperm parameter assessments

Whole sperm parameters (viability, motility, count, and normal morphology) decreased significantly (*p <* .05) after DM induction in the DM group than the control. Also, following administration of AS in both doses of 100 and 300 mg/kg in the DM + As groups, the sperm viability, motility, count, and normal morphology were increased significantly (*p <* .05) than in the control group. In DM + AS 500 mg/kg animals, only the count factor showed significant (*p <* .05) incremental changes than the DM group (Table 2).

**TABLE 2:** Effects of DM and AS administration on sperm parameters.

Experimental groups	Viability (%)	Motility (%)	Count (10^6^)	Normal morphology (%)
Control	74.83 ± 1.7	20.33 ± 1.8	88 ± 1.4	53.1 ± 0.7
DM	35.5 ± 0.7 ȸ	8 ± 0.2 ȸ	32.18 ± 0.7 ȸ	18.42 ± 0.5 ȸ
AS 100 mg/kg	73.2 ± 0.7	19.2 ± 0.8	86 ± 1.8	53.4 ± 0.8
AS 300 mg/kg	77.6 ± 0.9	21 ± 1.7	87 ± 1.9	52.9 ± 0.6
AS 500 mg/kg	75.2 ± 1.01	20.23 ± 1.1	86 ± 0.3	53.4 ± 0.4
DM + AS 100 mg/kg	54.83 ± 0.6 §	12.33 ± 0.4 §	80.5 ± 1.08 §	33.6 ± 0.4 §
DM + AS 300 mg/kg	65.34 ± 0.4 §	18 ± 0.7 §	85.7 ± 0.01 §	48.64 ± 0.3 §
DM + AS 500 mg/kg	36.3 ± 0.6	9.5 ± 0.5	80.6 ± 0.4 §	19.7 ± 0.14

*Note*: Data were analyzed using the Tukey's post hoc test and are presented as mean ± SD. ȸ indicates *p <* .05 compared with the control group, and § represents *p <* .05 compared with the DM group. *N* = 6 animals in each group.

Abbreviations: AS, aubergine stem; DM, diabetes mellitus.

### 3.4 Serum levels of fast blood sugar (FBS), Tes, LH, and FSH

After STZ administration, the FBS factor increased significantly (*p <* .05) in diabetic animals compared with the control group. Also, in whole doses of AS (100, 300, and 500 mg/kg), the FBS was decreased significantly (*p <* .05) in the DM + AS groups in comparison with DM animals. Tes and FSH levels were also decreased significantly (*p <* .05) after DM induction in the DM group compared with the control animals. Also, following administration of AS extract in whole groups of DM + AS 100, 300, and 500 mg/kg, the serum levels of Tes and FSH were significantly higher (*p <* .05) than in the DM group. Besides, LH levels were decreased in these groups following DM induction in comparison with the control animals, and this value was increased (to the physiological levels) in DM + AS 100, 300, and 500 mg/kg than the DM group (Table 3).

**TABLE 3:** Effects of DM and AS on various body parameters in male mice.

Experimental groups	FBS (ng/ml)	Tes (ng/ml)	LH (ng/ml)	FSH (ng/ml)	TAC (mmol/ml)	MDA (nm/gKW)	NO (OD)
Control	93.63 ± 0.4	9.5 ± 1.3	2.1 ± 0.2	35.2 ± 4.7	1.1 ± 0.04	61.84 ± 0.52	28.5 ± 1.2
DM	320.5 ± 0.7 ȸ	2.33 ± 0.6 ȸ	12.2 ± 1.4 ȸ	6.2 ± 1.0 ȸ	0.52 ± 0.08	114.1 ± 2.54 ȸ	48 ȸ
AS 100 mg/kg	92.36 ± 0.3	8.3 ± 1.1	1.9 ± 0.3	34.2 ± 3.9	1.32 ± 0.05	61.3 ± 2.03	31 ± 0.6
AS 300 mg/kg	93.12 ± 0.01	9.9 ± 0.3	2.4 ± 0.5	33.6 ± 2.9	2.33 ± 0.6 ȸ	60.6 ± 3.13	28.01 ± 1.1
AS 500 mg/kg	92.93 ± 0.1	8.5 ± 0.8	1.8 ± 0.8	34.6 ± 3.1	1.19 ± 0.13	60.4 ± 0.83	27.02 ± 2.2
DM + AS 100 mg/kg	112.83 ± 0.6 §	7.5 ± 0.4 §	6.2 ± 0.9 §	28.3 ± 2.6 §	1.78 ± 0.03 §	95.73 ± 2.98 §	30 ± 2.01 §
DM + AS 300 mg/kg	105.6 ± 0.8 §	8.76 ± 0.5 §	4.1 ± 0.1 §	29.7 ± 3.5 §	1.62 ± 0.03 §	94.8 ± 0.47 §	29.6 ± 1.6 §
DM + AS 500 mg/kg	121 ± 0.6 §	6.8 ± 0.4 §	3.7 ± 0.7 §	30.2 ± 3.9 §	1.01 ± 0.12 §	93.3 ± 5.3 §	32.3 ± 1.8 §

*Note*: Data were analyzed using the Tukey's post hoc test and are presented as mean ± SD. ȸ indicates *p <* .05 compared with the control group, and § represents *p <* .05 compared with the DM group. *N* = 6 animals in each treatment group.

Abbreviations: AS, aubergine stem; DM, diabetes mellitus; FBS, fast blood sugar; MDA, malondialdehyde; NO, nitric oxide; OD, optical density; TAC, total anti‐oxidant capacity; Tes, testosterone.

### 3.5 Serum levels of TAC, MDA, and NO

A significant (*p <* .05) increase in TAC level was detected in the AS 300 mg/kg group compared with control animals. Also, in whole doses of DM + AS 100, 300, and 500 mg/kg, a significant (*p <* .05) increase in TAC levels was found in comparison with the DM group. MDA value was found decreased in DM + AS 100, 300, and 500 mg/kg groups compared with DM animals, and also this value was significantly increased in the DM group compared with the control groups (*p <* .05). NO was increased significantly (*p <* .05) in the DM group compared with the control groups. This value showed a significant decremental trend in whole doses of DM + AS (100, 300, and 500 mg/kg) compared with the DM group (*p <* .05) (Table 3).

### 3.6 Histopathological assessments of testes tissue using H&E staining

**FIGURE 1:**
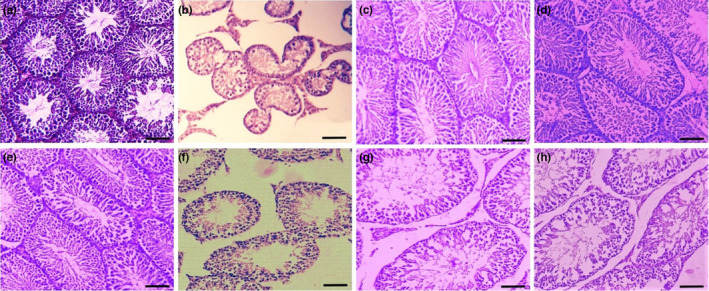
Histopathological H&E images (100×, scale bar: 200 μm) of testes in different groups of control (a), DM (b), AS 100 mg/kg (c), AS 300 mg/kg (d), AS 500 mg/kg (e), DM + AS100mg/kg (f), DM + AS300mg/kg (g), and DM + AS500mg/kg (h). *N* = 6 animals in each treatment group. AS, Aubergine stem; DM, diabetes mellitus.

Based on the quantitative findings, the whole parameters of seminiferous tubules diameter, germinal epithelium diameter, seminiferous tubules count, and number of sertoli cells were decreased significantly (*p <* .05) in DM animals compared with the control groups. Also, the assessments indicated that these factors were increased significantly (*p <* .05) in the DM + AS 300 and 500 mg/kg groups compared with DM animals (Table 4, Figure 1).

**TABLE 4:** Effect of DM and AS on various parameters of seminiferous tubules in male mice.

Experimental groups	Diameter of seminiferous tubule (μm)	Diameter of germinal epithelium (μm)	Number of seminiferous tubules	Number of sertoli cells
Control	598.3 ± 22.4	251.98 ± 18.5	13.2 ± 2.06	11.4 ± 1.52
DM	223.8 ± 17.7 ȸ	82.43 ± 12.6 ȸ	6.2 ± 1.8 ȸ	5.1 ± 0.5 ȸ
AS 100 mg/kg	563.34 ± 19.3	212.5 ± 15.1	12.2 ± 1.45	11.4 ± 1.03
AS 300 mg/kg	593.22 ± 18.91	263.9 ± 25.3	12.483 ± 1.67	12.06 ± 0.01
AS 500 mg/kg	582.43 ± 21.16	248.5 ± 18.8	13.9 ± 2.03	11.5 ± 0.8
DM + AS 100 mg/kg	245.83 ± 23.5	88.4 ± 17.4	7.8 ± 2.3	5.37 ± 0.8
DM + AS 300 mg/kg	525.4 ± 25.8 §	193.76 ± 20.7 §	11.25 ± 1.93 §	9.7 ± 0.47 §
DM + AS 500 mg/kg	509 ± 21.7 §	172.5 ± 18.8 §	11.31 ± 2.02 §	8.6 ± 0.6 §

*Note*: Data were analyzed using the Tukey's post hoc test and are presented as mean ± SD. ȸ indicates *p <* .05 compared with the control group and § represents *p <* .05 compared with DM group. N = 6 animals in each treatment group.

Abbreviations: AS, aubergine stem; DM, diabetes mellitus.

### 3.7 Assessment of apoptotic index (AI) using the TUNEL assay

**FIGURE 2:**
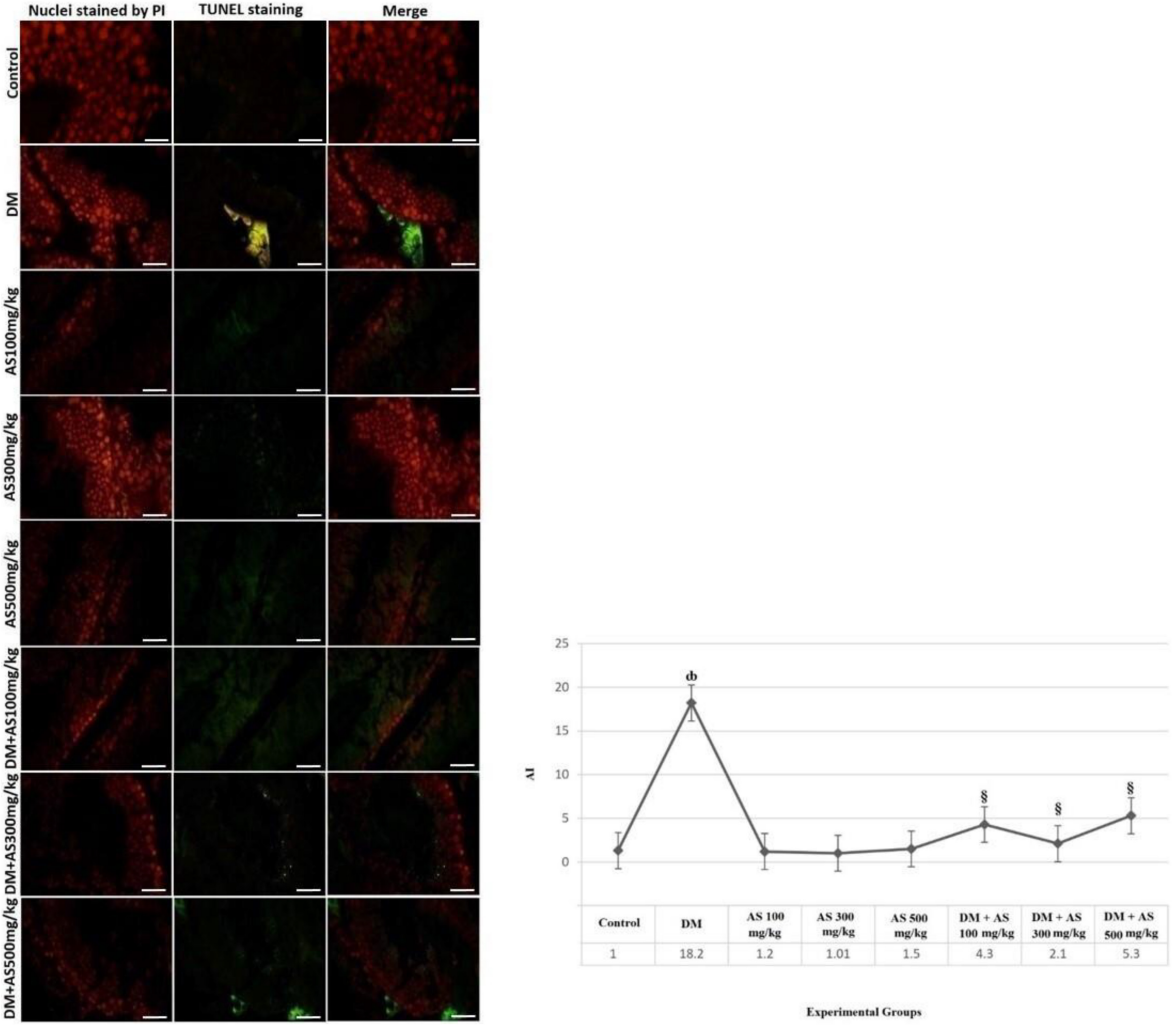
The TUNEL assay resultant on seminiferous tubular cells (400×) and the diagram of AI in experimental groups of control, DM, AS, and DM + AS with various doses of AS (100, 300, and 500 mg/kg). ȸ indicates *p <* .05 compared with the control group, and § represents *p <* .05 compared with the DM group. *N* = 6 animals in each treatment group. AI, apoptotic index; AS, aubergine stem; DM, diabetes mellitus.

AI was increased significantly (*p <* .05) in the DM group than in the control animals. This index was also decreased significantly (*p <* .05) in whole doses of AS (100, 300, and 500 mg/kg AS) in the DM + AS groups in comparison with the DM group represented in Figure 2.

### 3.8 Total body and testis weight

Total body weight and the weight of the testis decreased significantly in the DM group compared with the control animals (*p <* .05). Also, following administration of AS, the body weight and testis weight were increased significantly (*p <* .05) in DM + AS 300 mg/kg and DM + AS 500 mg/kg compared with the control group (Table 5).

**TABLE 5:** Effect of DM and AS on total body weight and testis in male mice.

Experimental groups	Total body weight (g)	Testis weight (g)
Control	25.3 ± 1.8	0.08 ± 0.01
DM	16.8 ± 0.7 ȸ	0.01 ± 0.001 ȸ
AS 100 mg/kg	25.4 ± 2.3	0.07 ± 0.02
AS 300 mg/kg	23.1 ± 1.9	0.09 ± 0.03
AS 500 mg/kg	22.3 ± 2.06	0.08 ± 0.008
DM + AS 100 mg/kg	17.3 ± 2.8	0.04 ± 0.03
DM + AS 300 mg/kg	25.2 ± 1.9 §	0.07 ± 0.06 §
DM + AS 500 mg/kg	26 ± 1.05 §	0.09 ± 0.03 §

*Note*: Data were analyzed using the Tukey's post hoc test and are presented as mean ± SD. ȸ indicates *p <* .05 compared with the control group and § represents *p <* .05 compared with DM group. *N* = 6 animals in each treatment group.

Abbreviations: AS, aubergine stem; DM, diabetes mellitus.

### 3.9 Levels of p53, Bcl‐2, and caspase‐3 gene expression

**FIGURE 3:**
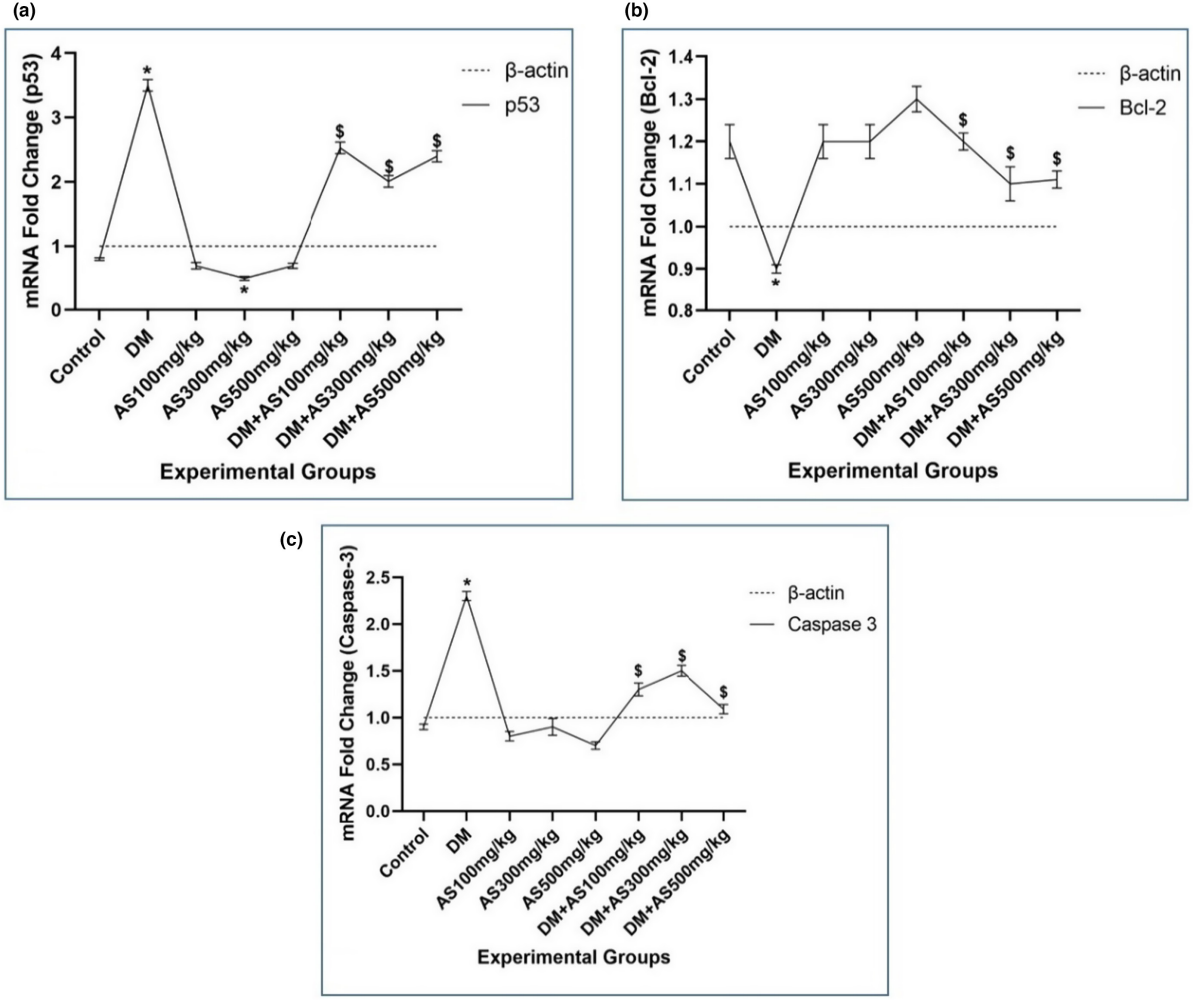
Gene expression of p53 (a), Bcl‐2 (b), and Caspase‐3 (c) in various treatment groups of control, DM, AS, and DM + AS (100, 300, and 500 mg/kg). * indicates *p <* .05 for the control group, and § represents *p <* .05 for the DM group. *N* = 6 animals in each treatment group. DM; diabetes mellitus; AS; Aubergine stem.

DM increased gene expression of p53 and caspase‐3 in the DM group compared with control animals significantly (*p <* .05). Bcl‐2 showed a significant decremental trend in the DM group compared with the control group (*p <* .05). In AS treatment groups, only p53 gene expression in the AS 300 mg/kg group represented significant (*p <* .05) decreased levels compared with the control group. In whole doses of AS (100, 300, and 500 mg/kg) in DM + AS groups, significant (*p <* .05) decremental changes were observed in p53 and caspase‐3 gene expression, and significant (*p <* .05) incremental alteration was also found in Bcl‐2 gene expression compared with the DM group (Figure 3).

## 4 DISCUSSION

DM is associated with decreased insulin receptor sensitivity located at the cell surface. As glucose is the main energy source in cells, the presence of extensive metabolic cellular changes is probable following the absence of these types of receptors. Besides, lipid or protein metabolism instead of glucose can increase oxidative levels abnormally. Thus, DM is associated with cellular metabolic changes, which eventually lead to increased levels of reactive oxygen specious (ROS) and reduced activity of intrinsic cellular antioxidant systems (Oguntibeju, 2019). One of the organs sensitive to oxidative stress is the reproductive system, in which the smallest changes in reproductive homeostasis can lead to major alterations in count and quality of sperm production. In the meantime, any type of factor with antioxidant properties can reduce the level of active radicals and contribute to increasing a cell's natural antioxidant system. Due to the antioxidant properties of AS, this herb can probably restore the normal function of the sexual system and the production of healthy sperm against the destructive effects of DM. In this study, we first investigated the probable antioxidant properties of AS (through phytochemical screening and FRAP assays) and also demonstrated the definite destructive effects of DM with laboratory protocols (through MDA and Griess assays). Finally, we evaluated tissue changes in DM animals following AS administration, genetically, histologically, and serologically. In the present research, we aimed to determine the restorative potential of AS extract against the destructive effects of DM in male NMRI mice.

The stem of eggplant is the green part of this vegetable. Although this part of eggplant is not edible, in traditional Iranian medicine, the AS was boiled in water, and the resulting solution was prescribed for DM patients. The phytochemical studies of AS showed that the majority of this stem was rich in substances with strong antioxidant properties, including saponins, flavonoids, and tannins. At the next level, there were fewer useful antioxidants, including alkaloids, phlobatannins, and anthraquinones. Antioxidants are chemicals with potential inhibitory effects on the oxidation process. During oxidation, ROS and free radicals attack many cellular macromolecules, which finally disrupt normal cell structures and physiological hemostasis. Based on their biochemical structure, there are two main types of antioxidants: hydrophilic and lipophilic. All types of antioxidants react with oxidants; water‐soluble and lipid‐soluble antioxidants react with cytosolic oxidants and cell membranes, respectively. These compounds may be synthesized in the body or obtained from the diet. As the phytochemical analysis of AS showed, it can be concluded that the main therapeutic properties of AS are due to its antioxidant properties. Although the presence of herbal antioxidant agents in AS was confirmed, the presence of active concentrations of antioxidant levels in serum seems more important. Thus, in the present study, the FRAP assay was also hired to measure TAC levels in blood serum after AS administration. The results of TAC measurements showed that AS with an active dose of 300 mg/kg can increase the TAC index more than the control group. Also, in whole doses of AS in the DM + AS groups, the TAC levels were higher than in the DM groups. These findings approved the antioxidant characteristics of AS in serum. This finding was also approved by Okmen B., who reported high levels of antioxidant agents in eggplant. This important note, the presence of agents with antioxidant properties, is probably considered as the reason for the many therapeutic features of AS. We also found the antioxidative properties of eggplant, as already found by Okmen et al. (2009). In this regard, an experimental study was conducted about the protective effects of eggplant against oxidative DNA damage in human lymphocytes using the single‐cell gel electrophoresis assay.

The treatment cells with eggplant extracts prevented DNA damage in human lymphocytes in response to hydrogen peroxide (Sukprasansap et al., 2019). Thus, as we reported in the present study, they also found that eggplants seem to be safe for consumption, and their extracts could protect against DNA damage. This finding was also parallel to our TUNEL results, in which a lower number of apoptotic cells were detected following AS administration in the seminiferous tubules of DM animals. In DM, various molecular pathways are activated, like advanced glycation end‐product (AGE) and protein kinase C. The activation of these molecular pathways are identified as pro‐oxidative processes (Ighodaro, 2018).

In this study, in order to assess the role of DM in increased levels of oxidative stress and the effects of AS in reducing ROS levels, the samples were analyzed biochemically using two main assays, including MDA and Griess. We found increased levels of MDA as a lipid peroxidation marker in the DM group than in the control group. This finding approved the oxidative property of DM on the animal body. Lipid peroxidation is the chain of reactions of oxidative degradation of lipids. It is a process in which free radicals receive electrons from lipids located in cell membranes, which is called ROS attack to the cell and organelle membrane. This abnormal process results in cell damage. It often affects polyunsaturated fatty acids because they contain multiple double bonds between methylene bridges, which possess reactive hydrogen atoms. The end products of lipid peroxidation are reactive aldehydes, such as MDA and 4‐hydroxynonenal. Thus, we found higher levels of MDA in the blood serum than the control group, representing high levels of lipid decomposition. Also, as stated previously, AS was potentially able to decrease the oxidation levels. Thus, we found low levels of MDA in DM animals following AS administration. This finding represented the antioxidant property of AS against high levels of oxidation following DM induction. Some studies have revealed the antioxidant features of plants. They indicated that anti‐oxidative agents with an herbal basis can potentially accelerate cellular antioxidant power, leading to decreased levels of DM in serum (Fauziah et al., 2020).

In conditions of inflammation, NO, in conjunction with other ROS, contributes to oxidative stress (Lubos et al., 2008). In this study, we also analyzed NO levels as a marker of oxidative stress along with MDA. Oxidative stress causes a complex dysregulation of cell metabolism and homeostasis. Following DM, the production of NO reaches higher levels than in physiologic conditions. These elevated levels of NO are produced by various types of cells throughout the body, which finally access the reproductive system through the bloodstream to induce organ damage (Pitocco et al., 2010). In the present study, we also found high levels of NO in serum following DM induction. As we concluded previously, AS with antioxidant activity can induce ameliorative effects, leading to decreased levels of NO in DM + AS groups. Although all doses of AS (100, 300, and 500 mg/kg) showed significant decremental changes in NO levels in the DM + AS groups, the differences were more obvious in AS with an effective dose of 300 mg/kg.

All efforts to evaluate a drug's efficacy on reproductive system function must ultimately reflect the quality of sperm parameters. This means that with no changes in sperm quality, the drug seems not applicable. For this purpose, to evaluate the effectiveness of AS on the repair of damage caused by diabetes, we also examined sperm parameters. Sperm based on some special conditions are susceptible to ROS attack: a low amount of cytoplasm, a low concentration of antioxidant enzymes, and a haploid chromosome number. Thus, detrimental damage to the sperm plasma membrane and DNA fragmentation (in the nuclear and mitochondrial genomes) are probable. Damaged plasma membranes can lead to the production of immotile sperm, and damaged DNA can induce apoptosis in sperm. Also, ROS attack on the sperm cell membrane induces the peroxidation of unsaturated fatty acids. Deformed membrane lipids cause abnormal fertilization leading to infertility. High levels of oxidative stress can potentially damage the cell membrane throughout lipid peroxidation reaction. In this molecular attack, the structural and dynamic features of the sperm cell membrane could be changed, leading to deformed sperm morphologically (Van der Paal et al., 2016). As we found in the present study, the percentage of normal sperm morphology decreased following DM induction. This index was also repaired after 4 weeks of AS administration in the DM + AS animal groups. Although in the control group, ~50% of all sperm had abnormal morphology, in the DM group, the percentage of sperm morphology decreased significantly. After AS administration, in both doses of 100 and 300 mg/kg AS, the percentage of normal sperm morphology increased. This finding was also supported by the study of Yelumalai and a coworker. They concluded that quercetin, as an herbal flavonol, can ameliorate sperm oxidative stress, leading to normal sperm morphology (Yelumalai et al., 2019). Sperm motility, an important index of male fertility, was also reduced in DM animals. This feature was resolved in the DM + AS 100 and 300 mg/kg groups. Any presence of ROS in reproductive tracts is associated with infertility. Although ROS at specific doses is crucial for the physiological functions of cells, high levels of this agent can interfere with the cellular antioxidant defense system and cell membranes, leading to variations in sperm parameters, especially motility. Lipid peroxidation can cause dramatic remodeling of the biochemical composition and biophysical properties of cellular and organelle membranes. Following lipid peroxidation in the mitochondrion, various detrimental consequences occur, including dissipation of membrane potential, electron leakage, increased ROS production, and reduced capacity for energy production (Nowicka‐Bauer & Nixon, 2020). These above‐mentioned pathologic cascades are what happens in DM. ROS disrupts the fluidity of lipids in the plasma membrane. Damaged membranes can affect sperm motility and sperm–oocyte fusion during fertilization. Besides, the damaged sperm cannot repair these pathologic changes due to insufficient cytoplasmic enzymes (Laleethambika et al., 2019). As we found increased levels of apoptosis in the spermatogenic lineage, the rate of viability and eventually the number of sperm decreased significantly in the DM group. Through the preparation of exogenous administration of AS, high levels of antioxidants were provided for sperm and spermatogenic cell lines to repair the induced damages following DM. Thus, in the present study, we found higher grades of viability index and cell count following AS administration in DM + AS groups than in DM animals. Although we found increased levels of sperm viability index in groups of DM + AS 100 mg/kg and DM + AS 300 mg/kg, no significant changes were found in the DM + AS group with a dose of 500 mg/kg in sperm viability. Besides, the sperm count was increased in whole groups of DM + AS 100, 300, and 500 mg/kg. We found no scientific justification for significant changes in sperm count and non‐significant changes in sperm viability in animals in the DM + AS 500 mg/kg group.

Histopathological changes following induction of DM are categorized into two main groups: pathologic changes in interstitial tissue and pathologic alterations in seminiferous tubules. Based on the accelerated expression of genes involved in apoptosis, the connective tissue aggregation in the extracellular matrix was decreased, and apoptosis in Leydig cells was possible, leading to decreased levels of Tes production and secretion. Besides, gene assessments and TUNEL assays showed that apoptosis was also found in spermatogenic cell lines, leading to a decreased number of sperm generation. Leydig cells are involved in Tes production. As stated in a study, the Leydig cells are vulnerable to the oxidative stress produced by the DM condition. Wankeu‐Nya and colleagues, in an experimental study on Leydig cells of DM animals using the ultrastructure technique, concluded that the Leydig cells can potentially represent apoptosis after DM induction (Wankeu‐Nya et al., 2019). As we found in the present study, decreased density of interstitial tissue around seminiferous tubules indicated elevated levels of Leydig cell apoptosis, which was approved by decreased levels of Tes.

Apoptosis is a normal and physiological process in which damaged cells are induced to commit suicide. In this mechanism, various types of signaling molecules and gene expression exist, including Caspase‐3, Bcl‐2, and p53. DNA damage is broadly seen in DM cases in various tissues. In DM, the mitochondrial membrane is damaged, leading to the release of enzymes involved in energy production into the intracellular matrix. This important non‐physiological event is considered as a spark for apoptosis initiation. p53 is considered as a tumor suppressor agent that is activated following DNA damage. Activation of p53 can potentially induce the cleavage of caspase‐3. These events finally lead to an increased rate of apoptosis. In this process, Bcl‐2, as an inhibitory factor of caspase‐3, also showed a decremental trend. In the present study, apoptosis was induced in seminiferous tubules and interstitial tissue. Thus, we found elevated levels of p53 and Caspase‐3 gene expression and decreased levels of Bcl‐2. Various studies also reported activation of gene expression involving apoptosis following DM induction. Zhao and colleagues assessed the apoptosis process following curcumin extract administration in DM rats. They concluded that curcumin can prevent the accumulation of oxidative stress in cells and modulate the Bax/Bcl‐2‐mediated cell death pathway. This finding represents the effects of antioxidant agents with an herbal basis on apoptosis prevention (Zhao et al., 2017). This finding was also found in our study using the TUNEL assay. As we detected, following DM induction, the concentration of fragmented DNA was traced by the TUNEL immunoassay technique. Also, AS administration showed lower levels of DNA fragmentation, indicating the genoprotective effects of AS. Due to the preservation of sperm and other interstitial cells, the occurrence of apoptosis is rare, as we found in the DM + AS groups.

## 5 CONCLUSION

Pathologic conditions of DM are mainly related to the induction of an unbalanced cellular antioxidant system. DM increased serum and tissue levels of MDA and NO. These molecules, at high levels, can affect other long‐distance cells diversely. Besides, administration of an exogenous source of antioxidants with an herbal basis seems useful against DM side effects. AS was found to be an herb rich in antioxidant agents. Antioxidants react with macromolecule components, especially cell and organelle membranes. In this way, lipid peroxidation was inhibited and the cellular levels of ROS were decreased. Thus, apoptosis was restrained, and the spermatogenic cell lines and interstitial tissue were shifted to a normal and physiological condition, leading to increased sperm production with high quality and Tes generation by Leydig cells. In this experimental study, we found AS to be an effective herbal antioxidant to prevent seminiferous cell line apoptosis, Leydig cell prevention, and the establishment of antioxidant balance in the reproductive system. In this way, high‐quality sperm were produced following normal Tes levels.

## AUTHOR CONTRIBUTIONS

**Parsa Rostamzadeh:** Investigation (equal); methodology (equal); project administration (equal); resources (equal). **Vahid Shokri‐Asl:** resources (equal); methodology (equal). **Fatemeh Mansouri Torghabeh:** Data curation (equal); methodology (equal); software (equal). **Samira Davoudi:** Software (equal); validation (equal). **Ahmad Haghzadeh:** Investigation (equal). **Shima Moradi:** Conceptualization (equal); project administration (equal); Data curation (equal); formal analysis (equal); writing – original draft (equal); writing – review and editing (equal).

## FUNDING INFORMATION

The authors received no financial support for the research, authorship, and publication of this article.

## CONFLICT OF INTEREST STATEMENT

The authors declare that they have no competing interests.

## Data Availability

The data that support the findings of this study are available from the corresponding author upon reasonable request.
